# ROBOTIC GASTRECTOMY: TECHNIQUE STANDARDIZATION

**DOI:** 10.1590/0102-672020200003e1542

**Published:** 2021-01-15

**Authors:** Andre Roncon DIAS, Marcus Fernando Kodama Pertille RAMOS, Daniel Jose SZOR, Ricardo ABDALLA, Leandro BARCHI, Osmar Kenji YAGI, Ulysses RIBEIRO-JUNIOR, Bruno ZILBERSTEIN, Ivan CECCONELLO

**Affiliations:** 1 Cancer Institute, Hospital das Clínicas, University of São Paulo, São Paulo, SP, Brazil

**Keywords:** Stomach neoplasms, Telesurgery, Robotic Surgical Procedures, Gastrectomy, Neoplasias gástricas, Telecirurgia, Gastrectomia, Robótica

## Abstract

**Background:**

Robotic gastrectomy is gaining popularity worldwide. It allows reduced blood loss and lesser pain. However, it widespread use is limited by the extensive learning curve and costs.

**Aim:**

To describe our standard technique with reduced use of robotic instruments.

**Methods:**

We detail the steps involved in the procedure, including trocar placement, necessary robotic instruments, and meticulous surgical description.

**Results:**

After standardizing the procedure, 28 patients were operated with this budget technique. For each procedure material used was: 1 (Xi model) or 2 disposable trocars (Si) and 4 robotic instruments. Stapling and clipping were performed by the assistant through an auxiliary port, limiting the use of robotic instruments and reducing the cost.

**Conclusion:**

This standardization helps implementing a robotic program for gastrectomy in the daily practice or in one`s institution.

## Graphical Abstract

[Fig f1a]

**Figure f1a:**
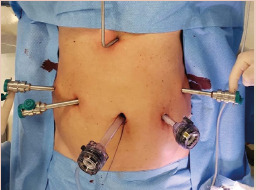
Trocars position for the Si model (position is similar for the Xi, although trocars stay more in line)

## RESUMO

**Racional:**

A gastrectomia robótica está ganhando popularidade no mundo. Ela permite menor perda sanguínea e menos dor. Entretanto, a curva de aprendizado extensa e os custos limitam seu amplo uso.

**Objetivo:**

Descrever nossa técnica padrão com uso reduzido de instrumental robótico.

**Métodos:**

Detalham-se todos os passos envolvidos no procedimento, incluindo posicionamento de portais, instrumentos robóticos necessários e descrição cirúrgica meticulosa.

**Resultados:**

Após a padronização do procedimento, 28 pacientes foram operados com essa técnica. Em cada procedimento foram utilizados: 1 (modelo Xi) ou 2 (Si) trocárteres descartáveis e 4 pinças robóticas. Grampeamentos e colocação de clips ficaram a cargo do auxiliar, reduzindo o número de instrumentos robóticos utilizados, diminuindo assim o custo.

**Conclusão:**

Essa padronização ajuda a implementar programa robótico de gastrectomia na prática diária ou em uma instituição.

## Graphical Abstract

[Fig f1as]

**Figure f1as:** Posição dos trocárteres para o sistema Da Vinci Si. Quando o modelo Xi é utilizado os portais são posicionados mais em linha reta.

## INTRODUCTION

Robotic gastrectomy in gastric cancer is gaining worldwide acceptance and studies are confirming its safety and efficacy. However, its widespread use is still limited due to costs and the necessity for multidisciplinary team massive training.

Laparoscopic gastrectomy has an extensive learning curve^6^and many differences when compared to the open approach: critical view, instrumental manipulation, presentation, ergonomics, etc. Robotic access is considered by some as an enhanced laparoscopy^11^, and those with experience in minimally invasive gastrectomy show quick adaptation^10, 12^. However, the access has its own particularities, with increased complications in the beginning and massive learning curve, despite laparoscopic expertise^8^.

Therefore, standardization is a key element to help implement robotic gastrectomy with reduced risk for patients. Alternatives to limit the cost are also desirable, especially in developing countries.

So, the objective of this article was to present a budget standardized technique for robotic D2 gastrectomy, using the Da Vinci system (Intuitive).

## METHODS

### Technique

#### Robotic material

1) One 12 mm disposable trocar (long, only for DaVinci Si Model); 2) one 12 mm disposable trocar (short); 3) three robotic 8 mm trocars (4 if Xi model); 4) one fenestrated bipolar forceps (robotic) or Maryland bipolar; 5) one harmonic scalpel (robotic); 6) one Cadiere (robotic); 7) one large needle driver (robotic)

#### Positioning

**FIGURE 1:**
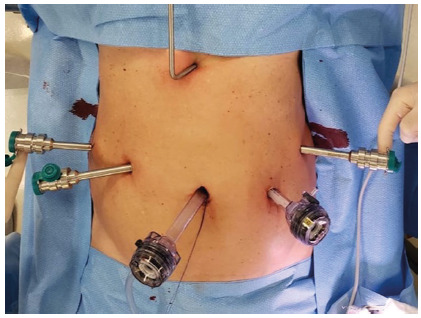
Trocars position for the Si model (position is similar for the Xi, although trocars stay more in line)

Patient is placed in supine position with 15^o^reverse Trendelenburg. For cavity access and optics, a supraumbilical incision is made and pneumoperitoneum established with 12 mmHg pressure. Work trocars are placed as presented in Figure 1. Robotic arms 1 and 3 stay at the right side of the patient. Arm 2 and the assistant port are placed on the left side. The assistant is responsible for clipping and stapling, reducing the need for robotic instruments. An epigastric 5 mm incision is made and a liver retractor placed. The patient`s cart is docked from the head when using the Si model, while Xi can be docked by the patient`s right side leaving the head free.

Instrumentation is performed with the harmonic scalpel on arm 1, fenestrated (or Maryland) bipolar forceps on arm 2 and Cadiere on arm 3. The forceps and the harmonic can be switched as needed. Arm 1 is controlled by the surgeon`s right hand, 2 and 3 by his left hand. Trocar positioning is always checked for their remote center location.

#### The procedure

**FIGURE 2:**
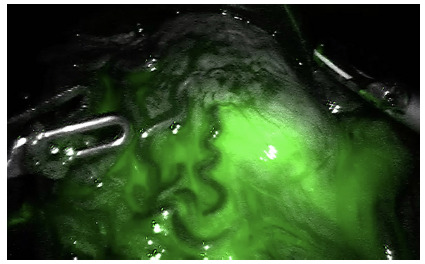
Proximal margin determined by indocyanine green fluorescence

Cavity is inspected and the tumor identified whenever possible. For early lesions whose location in the gastric body leaves doubt about the extent of the gastrectomy we suggest marking its borders preoperatively with indocyanine green (Figure 2), since intraoperative endoscopy increases the surgical duration and requires undocking if using the Si model.

The procedure starts by mobilizing the omentum. For advanced cases it is removed en-bloc with the specimen, while in early lesions the gastrocolic ligament is sectioned approximately 3 cm from the gastric arcade along the greater curvature.

Dissection is carried out in anti-clockwise fashion and the left gastro-epiploic vessels clipped and sectioned. For subtotal gastrectomy the greater curvature is prepared at least at the level of the first short gastric vessel (this may vary according to the lesion’s location and proximal margin required), while in total gastrectomy dissection stops after clearing the left diaphragmatic pilar.

Next, dissection goes clockwise until the pancreatic head and the duodenum are exposed. The pancreatic plateau is freed from the antrum and, whenever possible, lymph node station 8a dissected exposing the common hepatic artery. The gastroduodenal artery is dissected and the right gastroepiploic vessels clipped and sectioned, this clears lymph node station 6.

Dissection progresses to the suprapiloric region and after determining an adequate margin the duodenum is transected with a linear stapler operated by the assistant.

The hepatic hilum is cleared in its anterior aspect, removing station 12a and exposing the proper hepatic artery. Dissection is limited to the right by the bile duct. The procedure progresses clockwise with the stomach being pulled to the left of the patient. Dissection continues along the common hepatic artery and the left gastric vein is clipped and sectioned. Lymph node 11p is then removed en-bloc with the specimen.

**FIGURE 3:**
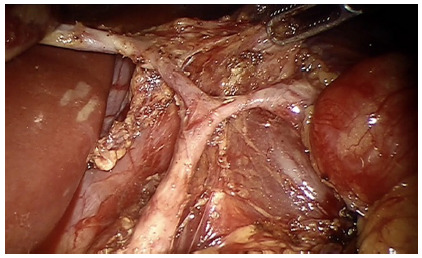
Artery from the celiac trunk branching into left gastric artery and left accessory hepatic artery

Station 9 is cleared; the gastrohepatic ligament divided and the left gastric artery sectioned after ligation with titanium or polymer clips (choice is based on its caliber). Nearly 10% of the patients have an accessory left hepatic artery (branch from the celiac trunk), it is spared whenever possible, removing the lymph nodes and dividing only the gastric branches (Figure 3).

For subtotal gastrectomy, the lesser curvature is cleared from stations 1 and 3 and the stomach sectioned with linear stapler. Reconstruction is performed in Roux-en-Y. The jejunum is then divided approximately 15-20 cm from the duodenojejunal flexure (Treitz) and an antecolic gastrojejunal anastomosis made with linear stapler on the gastric posterior wall. The stapler`s entry hole is closed in one plane of running suture with 3-0 polydioxanone. Transmesocolic fashion is chose when tension is observed or when the colic mesentery is accidentally opened during dissection. The alimentary loop is left with 60 cm and a side-by-side anastomosis performed with the biliary jejunal loop.

For total gastrectomy*,*the lesser curvature is left intact and the esophagus dissected. Lymph node stations 11d, 10, 19, 20, 110 and 111 are only removed in selected cases^1^. Roux-in-Y reconstruction with transmesocolic fashion is performed. A side-by-side esophagojejunostomy is performed before sectioning both structures. This allows the stomach to be used for traction. A boogie calibrates the anastomosis and then the esophagus is sectioned by a stapler. The entry hole (partially closed when sectioning the esophagus) is closed by manual suture. Alternatively, the esophagus may be divided before the anastomosis and the entry hole may be stapled^3^. When more extensive esophageal margin is required, the esophagus is divided and manual end-to-side esophagojejunostomy performed in two planes (calibrated with a boogie). This anastomosis is always tested with methylene blue and mesenteric holes closed. Sixty centimeters below the esophagojejunostomy, a mechanical side-by-side enteric anastomosis is performed. The jejunum is divided and this anastomosis brought to the inframesocolic area. 

In both subtotal and total gastrectomy, the mesenteric defects are closed and a drain is left over the duodenum and near the gastrojejunostomy or esophagojejunostomy. Specimen is retrieved by extending the supraumbilical port (preferred option in those with umbilical hernia)^9^or by suprapubic incision. When margins require frozen section, the specimen is removed before reconstruction. Lymph node stations are carefully dissected in fresh and then fixed in Carnoy`s solution for 12-24 h^7^ ^,^ ^13^.

## RESULTS

Gastric cancer surgeons with laparoscopic experience and certified in robotic surgery extensively debated the instruments to be used and the technical approach. The first cases were performed in swine and then in patients under informed consent. 

After the procedure was standardized and the team acquainted with the method, the trocar placement and the surgical steps, 28 patients were operated in our institution. 

For each procedure, 4 robotic instruments and1 (Xi model) or 2 (Si) disposable trocars were used. The assistant performed all stapling and clipping through the auxiliary port. 

At this time, we cannot share the surgical results, since after standardization, all patients operated were included in an ongoing randomized trial comparing robotic with open gastrectomy (ClinicalTrials.gov Identifier: NCT02292914).

## DISCUSSION

Robotic access has the advantages of 3-D view, more degrees of movement and better ergonomics when compared to laparoscopy^2^ ^,^ ^4^ ^,^ ^14^. This translates in lesser blood loss and arguably in quicker and less painful recovery^5^. However, the learning curve, despite laparoscopic experience, and the cost are two main drawbacks that prevent robotic implementation.

Obviously, extensive experience with gastric cancer and massive team and individual acquaintance with the equipment are required not to jeopardize the oncological results or increase the complication rate when compared to open or laparoscopic approaches. Here we presented our standardized approach to help those interested in overcoming the difficult first steps, when small issues such as trocar placement may add considerable operative time or difficulty to the procedure. We also limit the number of robotic instruments used to allow a budget option, which is extremely important in countries such as Brazil.

The approach presented here is the result of our experience, first with laparoscopic surgery, then with robotic procedures in swine and, only then, in patients under informed consent. It evolved over the last six years, thanks also to group discussions and debates with international peers. 

## CONCLUSION

Standardization of the robotic gastrectomy for gastric cancer reduces operative duration and abbreviates the learning curve. The number of robotic instruments used can be limited, reducing cost without jeopardizing results.
